# Dr. George Dallas Barrett

**Published:** 1916-02

**Authors:** 


**Dr. George Dallas Barrett,** Dartmouth 1878, died at his home in Clyde, Dec. 23, of nephritis. He was Health Officer of the town of Galen.

